# The Contrasting Effects of Two Distinct Exercise Training Modalities on Exhaustive Exercise-Induced Muscle Damage in Mice May Be Associated with Alterations in the Gut Microbiota

**DOI:** 10.3390/ijms25147837

**Published:** 2024-07-17

**Authors:** Yong Zhang, Cong Wang, Hedong Lang, Hongtao Yu, Min Zhou, Xin Rao, Qianyong Zhang, Long Yi, Jundong Zhu, Mantian Mi

**Affiliations:** Chongqing Key Laboratory of Nutrition and Health, Research Center for Nutrition and Food Safety, Institute of Military Preventive Medicine, Third Military Medical University (Army Medical University), NO. 30th Gao Tan Yan Street, Shapingba District, Chongqing 400038, China; zhangyong_017@tmmu.edu.cn (Y.Z.); wangcc1314@126.com (C.W.); lang0401@tmmu.edu.cn (H.L.); yhtao0053@163.com (H.Y.); zm19910510@tmmu.edu.cn (M.Z.); rx0207@tmmu.edu.cn (X.R.); zqianyong023@tmmu.edu.cn (Q.Z.); amu_yilong@tmmu.edu.cn (L.Y.)

**Keywords:** exhaustive exercise, muscle damage, gut microbiota, short-chain fatty acids, inflammation, oxidative stress

## Abstract

Exhaustive exercise is known to induce muscle damage characterized by inflammation and oxidative stress. Although “regular” and “weekend warrior” exercise regimens have been shown to confer comparable health benefits in human studies, such as reduced risks of all-cause, cardiovascular disease (CVD), and cancer mortality, their differential impacts on muscle damage post-exhaustive exercise remain unclear. This study aimed to compare the effects of long-term, moderate-intensity (LTMI) and short-term, high-intensity (STHI) training modalities, matched for total exercise volume, on gut microbiota, short-chain fatty acids (SCFAs), and exhaustive exercise-induced muscle damage in mice, as well as to evaluate the correlation between these factors. LTMI is considered a regular exercise regimen, while STHI shares some similarities with the “weekend warrior” pattern, such as promoting exercise intensity and condensing training sessions into a short period. Our findings indicate that LTMI training significantly enhanced the abundance of SCFA-producing bacteria, including *Akkermansia*, *Prevotellaceae_NK3B31_group*, *Odoribacter*, *Alistipes*, and *Lactobacillus*, thereby increasing SCFA levels and attenuating muscle damage following exhaustive swimming. In contrast, STHI training increased the abundance of opportunistic pathogens such as *Staphylococcus* and *Bilophila*, without altering SCFA levels, and was associated with exacerbated muscle damage. Moreover, we observed a significant negative correlation between the abundance of SCFA-producing bacteria and SCFA levels with the expression of inflammatory cytokines in the muscle of mice post-exhaustive exercise. Conversely, the abundance of *Staphylococcus* and *Bilophila* showed a notable positive correlation with these cytokines. Additionally, the effects of LTMI and STHI on exhaustive exercise-induced muscle damage were transmissible to untrained mice via fecal microbiota transplantation, suggesting that gut microbiota changes induced by these training modalities may contribute to their contrasting impacts on muscle damage. These results underscore the significance of selecting an appropriate training modality prior to engaging in exhaustive exercise, with implications for athletic training and injury prevention.

## 1. Introduction

With the increasing recognition of the health benefits of exercise and a heightened awareness of the importance of challenging oneself, global participation in long-distance endurance events, such as marathons and half-marathons, has surged significantly in recent decades [1]. However, marathon running, being a prolonged strenuous exercise, can trigger significant increases in plasma levels of inflammatory cytokines, including Interleukin (IL)-6, IL-8, and Tumor Necrosis Factor alpha (TNF-α). This physical exertion can also result in skeletal muscle damage, evidenced by an increase in plasma creatine kinase (CK) and lactate dehydrogenase (LDH) activity, and myoglobin concentration. Additionally, myocardial injury may be inferred from the heightened levels of troponin and N-terminal pro-B-type natriuretic peptide (proBNP) in plasma. These physiological responses suggest that the substantial physical stress of marathon running has the potential to initiate a profound systemic inflammatory response and cause multi-organ impairment, notably affecting the heart and skeletal muscle [2,3,4]. Moreover, marathon running increases the risk of upper respiratory tract infection, according to a recent meta-analysis accepted for publication in the Exercise Immunology Review [5]. While regular exercise training, as practiced by professional runners, may mitigate the inflammation and organ damage caused by marathons [6,7], many amateur runners lack the time to engage in such training regimens.

For substantial health benefits, adults should aim for a minimum of 150 to 300 min per week of moderate-intensity aerobic exercise, or 75 to 150 min of vigorous-intensity aerobic exercise, or an equivalent combination of both. Ideally, aerobic activity should be evenly distributed throughout the week to maintain a regular exercise pattern and ensure a consistent level of physical engagement [8]. The “weekend warrior” exercise pattern, characterized by performing intense exercise in one or two sessions on the weekends, has become popular due to its time efficiency [9]. Recent evidence suggests that this condensed physical activity pattern may offer health benefits similar to those of regular exercise, such as reduced risks of all-cause, cardiovascular disease (CVD), and cancer mortality, despite requiring less time commitment [10,11,12]. In the context of marathon training, some amateur runners might engage in high-intensity training sessions for several consecutive days, a pattern that differs from the recommended regular distribution of exercise. While this approach shares some similarities with the “weekend warrior” pattern, such as increasing exercise intensity and compressing training sessions into a short period, it is uncertain whether the same health benefits comparable to those of regular exercise apply, particularly in terms of reducing inflammation and organ damage induced by subsequent exhaustive exercise. Further research is needed to understand the specific impacts of condensed versus spread-out exercise training in this context.

Exercise training has been shown to interact with gut microbiota [13]. Gut microbiota is influenced by both non-modifiable factors, such as gender, genetics, and age, as well as modifiable factors, including diet, physical activity, and diseases [14]. Emerging findings in human and animal studies have shown that exercise is one of the main modifiable factors affecting gut microbiota composition and function, with the impact varying by exercise intensity, duration, and frequency [15,16]. Concurrently, the host’s adaptation to exercise may be influenced by gut microbiota through regulation of metabolism, oxidative stress, and inflammatory response [17,18]. However, the specific effects of different training patterns with the same total volume of exercise on gut microbiota, and how these patterns subsequently influence the body’s adaptation to exhaustive exercise like marathons, remain to be fully elucidated. 

In this study, we aimed to determine the impact of two distinct training modalities on gut microbiota, short-chain fatty acids (SCFAs), and exhaustive exercise-induced muscle damage in mice. We also investigated whether changes in gut microbiota could influence the effects of exercise regimens on muscle damage post-strenuous activity. The modalities compared were a short-term, high-intensity (STHI) pattern and a long-term, moderate-intensity (LTMI) pattern, with equal total exercise volumes. LTMI represents a consistent, regular exercise routine, whereas STHI mirrors the “weekend warrior” approach by emphasizing intense workouts concentrated within a brief timeframe.

## 2. Results 

### 2.1. Effects of Two Different Training Modalities on Muscle Damage in Mice following Exhaustive Exercise

**Figure 1:**
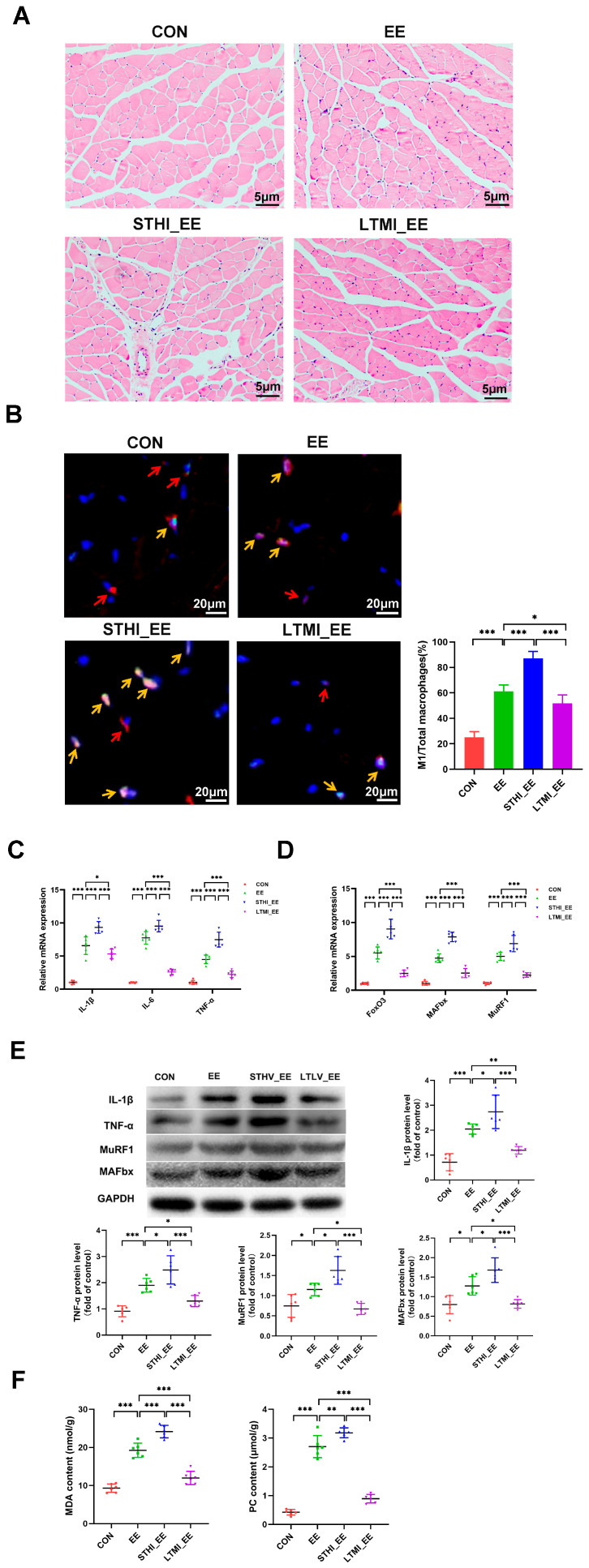
Effects of two different training modalities on muscle damage in mice submitted to exhaustive swimming. (**A**) representative images of H&E staining of gastrocnemius sections (*n* = 6 per group). (**B**) representative merged images of double immunofluorescence staining for *F4/80* (red) and *iNOS* (green) to respectively identify total macrophages and M1 macrophages in gastrocnemius sections. Red arrows indicate macrophages, yellow arrows indicate M1 macrophages. Bar graphs show quantification of M1-type macrophages (% of total macrophages in each field) in different groups. (**C**) mRNA levels of inflammatory cytokines *IL-1β*, *IL-6*, and *TNF-α* in gastrocnemius. (**D**) mRNA levels of muscle atrophy-related genes *FoxO3*, *MAFbx*, and *MuRF1* in gastrocnemius (*n* = 6 per group). (**E**) representative Western blots and quantified protein levels of IL-1β, TNF-α, MAFbx, and MuRF1 in gastrocnemius (*n* = 6 per group). (**F**) contents of oxidative stress biomarkers MDA and PC in gastrocnemius. Data are presented as means ± SD. Two-way ANOVA multiple comparison test was used (*n* = 6 per group). * *p* < 0.05, ** *p* < 0.01, *** *p* < 0.001. CON, sedentary group; EE, exhaustive exercise group; STHI_EE, exhaustive exercise after short-term, high-intensity training group; LTMI_EE, exhaustive exercise after long-term, moderate-intensity training group; H&E, Hematoxylin and Eosin; iNOS, inducible Nitric Oxide Synthase; IL-1β, Interleukin-1 beta; IL-6, Interleukin-6; *TNF-α*, Tumor Necrosis Factor alpha; FoxO3, Forkhead box O3; MAFbx, Muscle Atrophy F-box; MuRF1, muscle-specific RING Finger Protein 1; MDA, malondialdehyde; PC, protein carbonyl.

The effects of exhaustive swimming on muscle damage were assessed by examining morphological changes in the gastrocnemius muscle tissue of mice, stained with Hematoxylin and Eosin (H&E). Muscle fibers in the EE group showed disorganization and inflammatory cell infiltration, as compared with the control group. Mice subjected to LTMI training exhibited reduced muscle fiber disarray and decreased inflammatory cell infiltration, suggesting a protective effect against exhaustive swimming-induced muscle damage. Conversely, STHI training exacerbated these indicators of muscle damage (Figure 1A).

Consistent with histopathological observations, exhaustive swimming significantly increased the infiltration of M1 macrophages, as measured by immunofluorescence staining (Figure 1B), and enhanced expression of pro-inflammatory cytokines *IL-1β*, *IL-6*, and *TNF-α* at the mRNA level within the gastrocnemius muscle. LTMI training mitigated these inflammatory responses, while STHI training intensified them (Figure 1C). Protein levels of IL-1β and TNF-α paralleled the mRNA expression patterns, confirming the inflammatory trends (Figure 1E).

Given the link between inflammation and muscle atrophy, we evaluated the expression of atrophy-related genes *FoxO3*, *Atrogin-1*, and *MuRF1*. Exhaustive swimming significantly upregulated these markers, an effect that was attenuated in the LTMI-trained group and exacerbated in the STHI-trained group (Figure 1D,E).

Oxidative stress, indicated by elevated levels of malondialdehyde (MDA) and protein carbonyl (PC) in muscle, was significantly induced by exhaustive exercise. This oxidative stress response was notably reduced in mice undergoing LTMI training and further increased in those undergoing STHI training (Figure 1F).

In summary, the two training modalities markedly influenced muscle damage following exhaustive exercise, with LTMI training offering protective effects and STHI training potentially worsening muscle damage.

### 2.2. Effects of Two Different Training Modalities on the Levels of Serum CK, LDH, and BUN in Mice following Exhaustive Exercise

**Figure 2:**
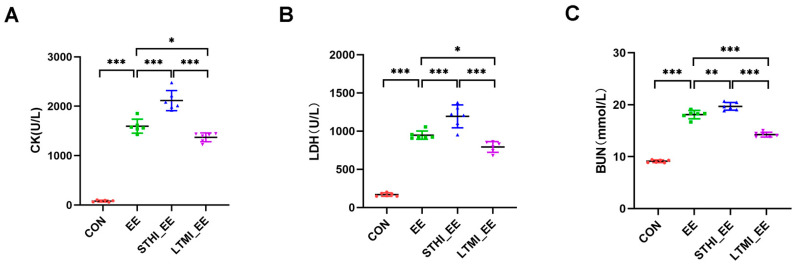
Serum levels of CK, LDH, and BUN in mice following exhaustive swimming. (**A**) Serum levels of CK; (**B**) serum levels of LDH; (**C**) serum levels of BUN. Each data point represents the mean ± standard deviation (SD) of the measured values. Inter-group comparisons were conducted using Student’s *t*-test, with statistical significance denoted by asterisks: * *p* < 0.05, ** *p* < 0.01, *** *p* < 0.001 (*n* = 6 per group). CON, sedentary group; EE, exhaustive exercise group; STHI_EE, exhaustive exercise after short-term, high-intensity training group; LTMI_EE, exhaustive exercise after long-term, moderate-intensity training group; CK, creatinine kinase; LDH, lactate dehydrogenase; BUN, blood urine nitrogen.

Beyond observing morphological changes indicative of tissue injury, the assessment of muscle damage induced by exhaustive exercise was further conducted by measuring alterations in the levels of serum creatinine kinase (CK), lactate dehydrogenase (LDH), and blood urine nitrogen (BUN), which are commonly recognized as biomarkers of muscle damage. The findings indicate that exhaustive swimming led to a significant rise in serum levels of CK, LDH, and BUN. Yet, this increase was notably attenuated in the LTMI training group, while it was further intensified in the STHI training group (Figure 2A–C). 

### 2.3. Effects of Two Different Training Modalities on Fecal Microbiota and Its SCFA Metabolites in Mice before Exhaustive Exercise

**Figure 3:**
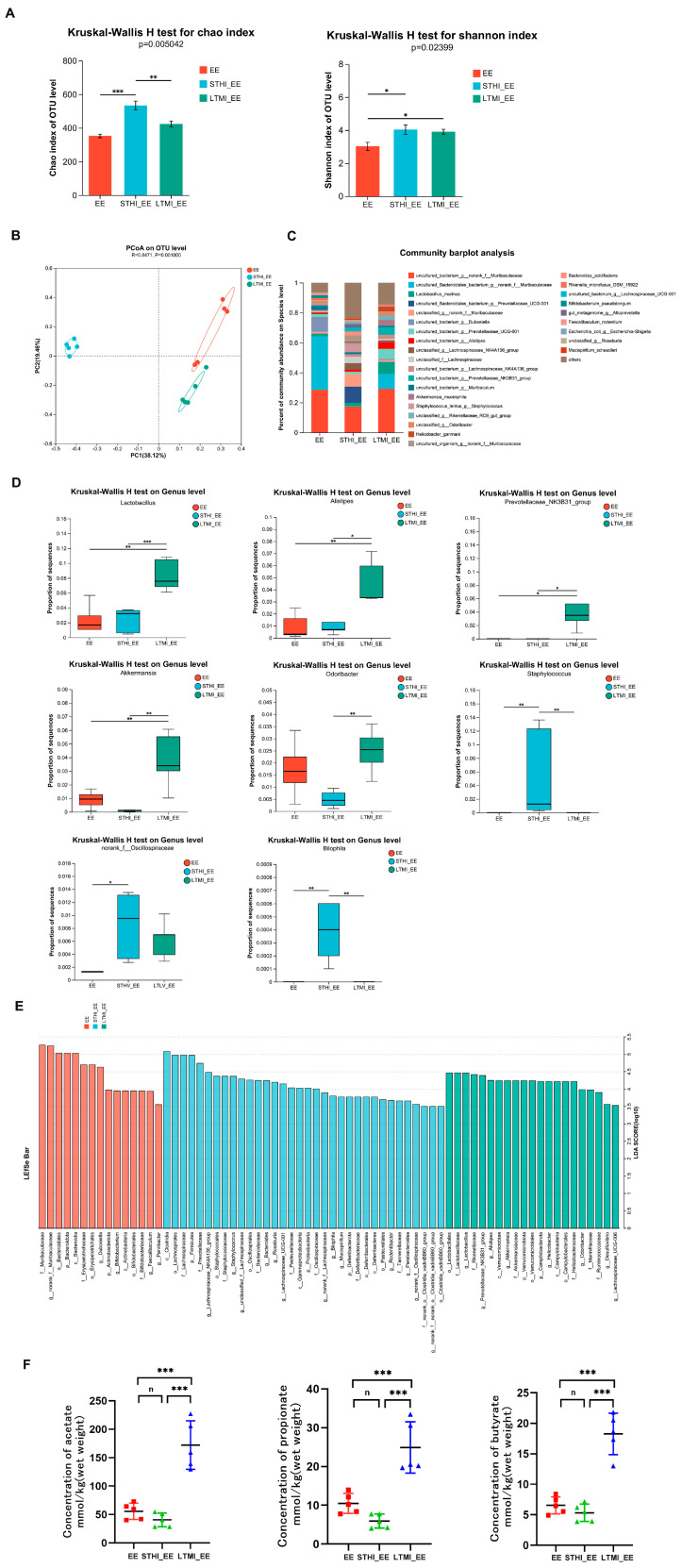
Effects of two different training modalities on gut microbiota composition and fecal SCFAs in mice before engaging in exhaustive exercise. (**A**) gut microbiota diversity using the Chao and Shannon indices (*n* = 5 per group). (**B**) a clear separation was observed by principal coordinate analysis (PCoA) based on Hellinger (*n* = 5 per group). (**C**) species–level bacterial community structure across different groups (*n* = 5 per group). (**D**) Kruskal–Wallis H test bar plot at the genus level (*n* = 5 per group). (**E**) bar graph of linear discriminant analysis (LDA) scores, showing a biomarker taxa (LDA score of >3.5 and a significance of *p* < 0.05 determined by the Wilcoxon signed–rank test) (*n* = 5 per group). (**F**) variations in the abundance of short–chain fatty acids. * *p* < 0.05, ** *p* < 0.01, *** *p* < 0.001. (Student’s *t*-test; n, no significance). EE, exhaustive exercise group; STHI_EE, exhaustive exercise after short–term, high–intensity training group; LTMI_EE, exhaustive exercise after long–term, moderate–intensity training group.

To explore the impact of various training regimens on gut microbiota, fecal samples were collected 24 h following the final training session. The V3 and V4 regions of the 16S rRNA variable genes were targeted for amplification and sequencing using the Illumina MiSeq platform. The α-diversity of the microbial communities, as gauged by the Chao and Shannon indices, exhibited significant variations in richness and diversity among the three groups (Kruskal–Wallis test, *p* < 0.05) (Figure 3A). Principal coordinate analysis (PCoA), based on the Bray–Curtis distances, clearly depicted a separation among the groups along the first ordination axis (PC1), which explained 38.12% of the observed variance (Figure 3B). The bacterial community structure at the species level for each group is depicted in Figure 3C. A deeper analysis at the genus level revealed that the LTMI group exhibited significantly increased relative abundance of *Lactobacillus*, *Alistipes*, *Prevotellaceae_NK3B31_group*, and *Akkermansia* compared to the EE group and STHI group, while the STHI group had a markedly increased relative abundance of *Staphylococcus* and *Bilophila* compared to the EE group and LTMI group (Figure 3D). Linear Discriminant Analysis (LDA) and effect size (LEfSe) analysis identified 70 differentially abundant taxa among the three groups, with an LDA score threshold > 3.5. The LTMI group demonstrated a significant presence of various microbial taxa, particularly those from the phyla *Verrucomicrobiota* and *Campilobacterota*. Additionally, several families stood out, including *Lactobacilliaceae*, *Rikenellaceae*, *Akkermansiaceae*, and *Marinifilaceae*. Noteworthy at the genus level are *Prevotellaceae_NK3B31_group*, *Akkermansia*, *Odoribacter*, *Alistipes*, and *Lactobacillus*, all of which were found in relatively high abundance. Conversely, the STHI group displayed a different microbial composition, with a notable increase in members from the phyla *Firmicutes* and *Proteobacteria*. Families such as *Lachnospiraceae*, *Prevotellaceae*, *Staphylococcaceae*, and *Bacteroidaceae* were particularly prominent within this group. At the genus level, *Lachnospiraceae_NK4A136_group*, *Staphylococcus*, *Bacteroides*, *Roseburia*, and *Bilophila* were identified as being relatively abundant (Figure 3E). Collectively, these findings underscore that distinct training modalities can significantly alter the composition of gut microbiota.

To further assess the impact of the two distinct exercise training regimens on gut microbiota, the levels of major short-chain fatty acids (SCFAs) produced by gut microbiota, including acetate, propionate, and butyrate, were measured in fecal samples. The results indicated that the LTMI training significantly increased fecal levels of acetate, propionate, and butyrate. However, the STHI training had no significant effects on the levels of these SCFAs (Figure 3F). 

The results collectively imply that two distinct training modalities exert divergent influences on gut microbiota and the production of SCFAs.

### 2.4. Analysis of the Correlations between Muscle Damage and Gut Microbiota at the Genus Level and Short-Chain Fatty Acids

**Figure 4:**
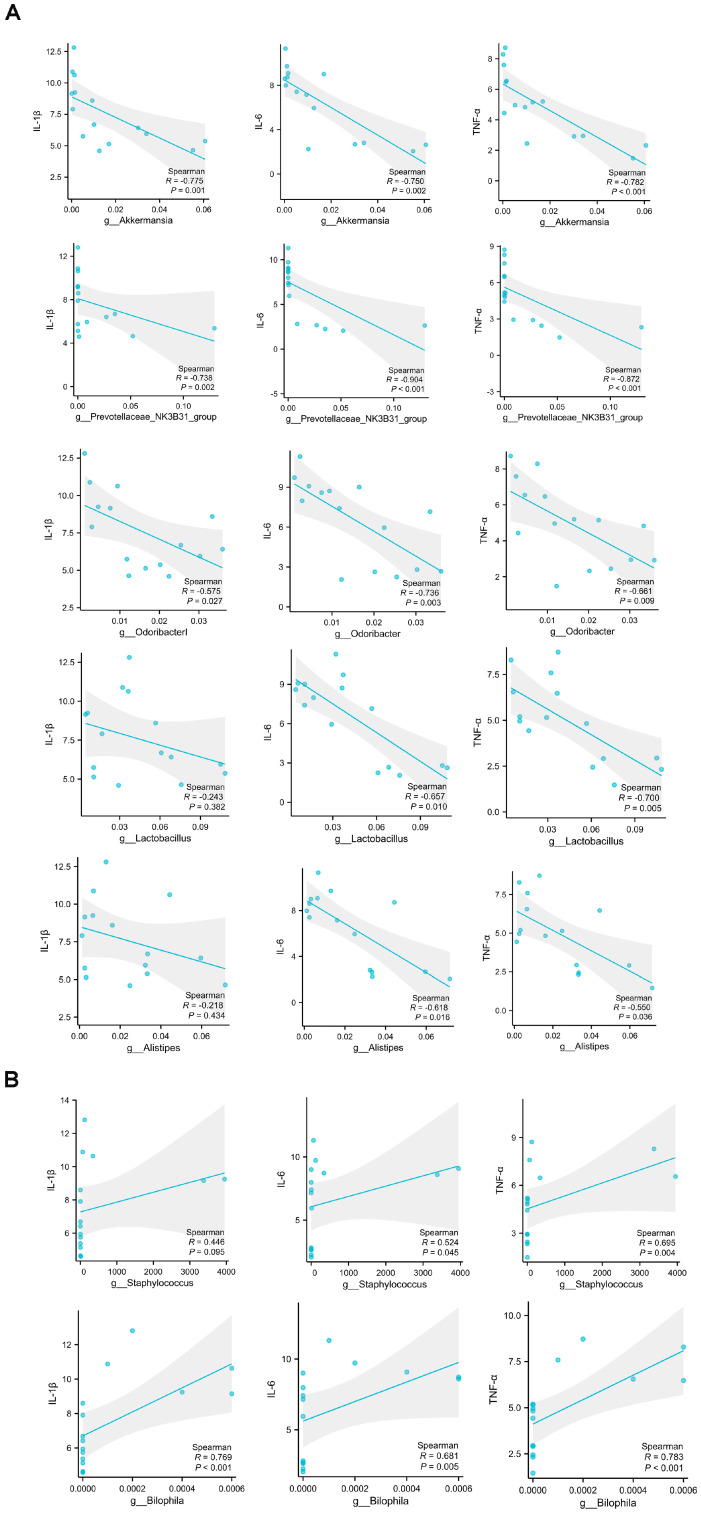
Correlation analysis of muscle damage indicated by the levels of pro–inflammatory cytokines expression and the gut microbial community at the genus level. (**A**) plots show the relationship between the level of *IL-1β*, *IL-6*, and *TNFα* mRNA and the abundance of *Akkermansia*, *Prevotellaceae_NK3B31_group*, *Odoribacter*, *Alistipes*, and *Lactobacillus*, represented as a smoothing spline with a 95% confidence interval (shaded region). (**B**) plots show the relationship between the level of *IL-1β*, *IL-6*, and *TNFα* mRNA and the abundance of *Staphylococcus* and *Bilophila*, represented as a smoothing spline with a 95% confidence interval (shaded region). ((**A**,**B**), Pearson’s correlation). *IL-1β*, Interleukin-1 beta; *IL-6*, Interleukin-6; *TNF-α*, Tumor Necrosis Factor alpha.

**Figure 5:**
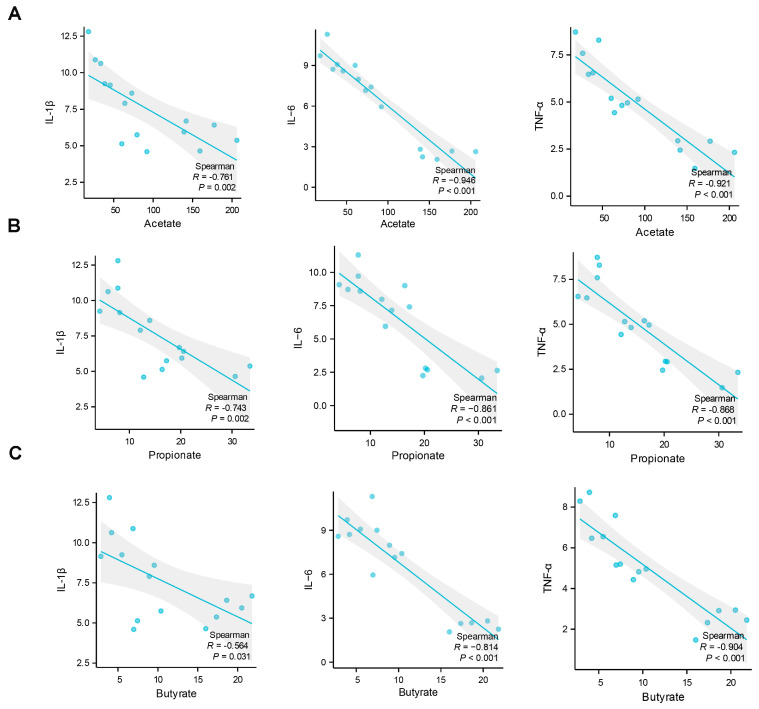
Correlation analysis of muscle damage indicated by the levels of pro-inflammatory cytokines expression and fecal SCFAs levels. (**A**–**C**) plots show the relationship between the level of *IL-1β*, *IL-6*, and *TNFα* mRNA and the content of acetate, propionate, and butyrate in feces, represented as a smoothing spline with a 95% confidence interval (shaded region). ((**A**–**C**), Pearson’s correlation). *IL-1β*, Interleukin-1 beta; *IL-6*, Interleukin-6; *TNF-α*, Tumor Necrosis Factor alpha.

In order to investigate the possible contributions of the alterations in gut microbiota to the differential effects of various exercise training regimens on muscle damage following exhaustive exercise, we conducted an analysis to determine the relationship between markers of muscle damage, as reflected by the expression levels of inflammatory cytokines, and the presence of specific gut microbial communities at the genus level, as well as the fecal levels of SCFAs. The findings revealed significant correlations between the expression levels of certain inflammatory cytokines and the abundance of specific gut microbial genera. Notably, the levels of *IL-6* and *TNFα* mRNA expression exhibited a strong negative correlation with the abundance of *Akkermansia*, *Prevotellaceae_NK3B31_group*, *Odoribacter*, *Alistipes*, and *Lactobacillus*. Additionally, the expression of *IL-1β* mRNA was notably and negatively associated with the abundance of *Akkermansia*, *Prevotellaceae_NK3B31_group*, and *Odoribacter* (Figure 4A). Conversely, the expression levels of *IL-1β*, *IL-6*, and *TNFα* mRNA were found to have a significant positive correlation with the abundance of *Staphylococcus* and *Bilophila* (Figure 4B). Further analysis indicated that the mRNA expression levels of *IL-1β*, *IL-6*, and *TNFα* were significantly and inversely associated with the fecal contents of acetate, propionate, and butyrate, as shown in Figure 5A–C. 

### 2.5. Effects of Fecal Microbiota Transplantation from Mice That Underwent Different Exercise Training Modalities on Exhaustive Exercise-Induced Muscle Damage in Recipient Mice

To ascertain the role of gut microbiota in the differential effects of various exercise training modalities on muscle damage from exhaustive exercise, we conducted a fecal microbiota transplantation (FMT) experiment. This experiment utilized fecal samples from mice that underwent different exercise training modalities, as described in study 1. After a 4-week FMT period, recipient mice completed an exhaustive swimming protocol, preceded by a 5-day adaptive swimming training phase to acclimate. Post-exhaustive swimming muscle damage indicators, as outlined in study 1, were assessed.

**Figure 6:**
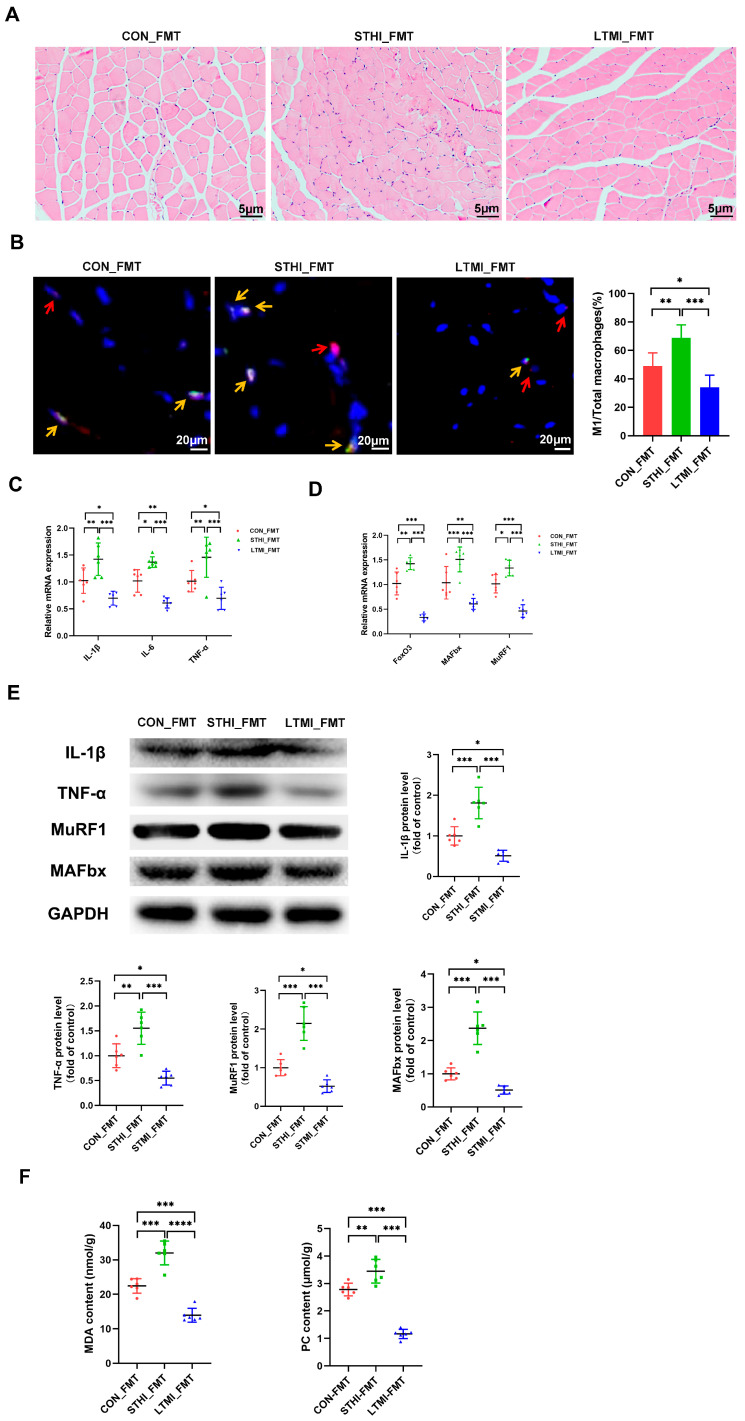
Effects of fecal microbiota transplantation from mice subjected to different exercise training modalities on muscle damage induced by exhaustive exercise. (**A**) representative images of H&E staining of gastrocnemius sections (*n* = 6 per group). (**B**) representative merged images of double immunofluorescence staining for *F4/80* (red) and *iNOS* (green) to respectively identify total macrophages and M1 macrophages in gastrocnemius sections. Red arrows indicate macrophages, yellow arrows indicate M1 macrophages. Bar graphs show quantification of M1-type macrophages (% of total macrophages in each field) in different groups. (**C**) mRNA levels of inflammatory cytokines *TNF-α*, *IL-1β*, and *IL-6* in gastrocnemius. (**D**) mRNA levels of muscle atrophy-related genes *FoxO3*, *MAFbx*, and *MuRF1* in gastrocnemius (*n* = 6 per group). (**E**) representative Western blots and quantified protein levels of *IL-1β*, *TNF-α*, *MuRF1*, and *MAFbx* in gastrocnemius (*n* = 6 per group). (**F**) the contents of oxidative stress biomarkers MDA and PC in gastrocnemius. Data are presented as means ± SD. Two-way ANOVA multiple comparison test was used (*n* = 6 per group). * *p* < 0.05, ** *p* < 0.01, *** *p* < 0.001, **** *p* < 0.0001. CON_FMT, transplantation of fecal microbiota of sedentary mice in study 1; STHI_FMT, transplantation of fecal microbiota of STHI training mice in study 1; LTMI_FMT, transplantation of fecal microbiota of LTMI training mice in study 1; H&E, Hematoxylin and Eosin; iNOS, inducible Nitric Oxide Synthase; IL-1β, Interleukin-1 beta; IL-6, Interleukin-6; TNF-α, Tumor Necrosis Factor alpha; FoxO3, Forkhead box O3; MAFbx, Muscle Atrophy F-box; MuRF1, muscle-specific RING Finger Protein 1; MDA, malondialdehyde; PC, protein carbonyl.

The LTMI_FMT group demonstrated protective effects against exhaustive swimming-induced muscle damage, similar to those observed in mice subjected to LTMI training. In contrast, the STHI-FMT group showed an exacerbation of muscle damage, consistent with the outcomes in mice that underwent STHI training (Figure 6A–F). These results indicate that gut microbiota alterations due to exercise training may play a significant role in the divergent impacts of these modalities on muscle damage following exhaustive exercise.

## 3. Discussion

In recent years, long-distance running, such as half-marathons and marathons, has rapidly gained popularity worldwide. However, mounting evidence indicates that prolonged strenuous exercise like long-distance running, as exhaustive exercise, can lead to increased oxidative stress and inflammatory responses in multiple organs, including skeletal muscle, heart, and liver, particularly among amateur runners. Prior to long-distance running participation, some amateur runners engage in LTMI training as a form of exercise preconditioning, while others, due to time constraints, adopt STHI training as a preconditioning strategy. However, the effectiveness of these two distinct exercise training modalities in protecting against prolonged strenuous exercise-induced muscle damage remains unclear. This study utilized a mouse model of exhaustive swimming to investigate the impact of these two different exercise training modalities on skeletal muscle damage caused by the subsequent exhaustive exercise and the role of gut microbiota in this context. The results revealed that LTMI training can alleviate exhaustive exercise-induced skeletal muscle damage, while STHI training may exacerbate such damage, with both opposing effects being associated with respective alterations in gut microbiota.

Exercise-induced muscle damage (EIMD) is characterized by muscle inflammation, oxidative stress, and elevated serum biomarkers such as myoglobin, CK, LDH, and BUN. Prolonged strenuous exercise, like marathon running and exhaustive treadmill running or swimming, can cause muscle damage in both human and animal models [19,20,21,22]. Exercise preconditioning is thought to mitigate EIMD by inducing a range of beneficial physiological adaptations. The WHO’s 2020 guidelines recommend 150 to 300 min of moderate-intensity or 75 to 150 min of vigorous-intensity aerobic activity per week, and that an equivalent combination of these two intensities is also acceptable. Preferably, aerobic activity should be spread throughout the week to maintain a regular exercise pattern [8]. Recent studies suggest that there may be no significant difference in health benefits between the same volume of physical activity condensed into one or two sessions per week, often referred to as the “weekend warrior” approach, and the same activity spread across multiple sessions throughout the week, known as the “regular exercise” pattern [23,24,25]. However, limited research has directly compared these two exercise patterns in terms of their impact on muscle damage following strenuous endurance events, such as marathon running. Our study found that exhaustive swimming caused muscle fiber disorganization, inflammatory cell infiltration, increased M1 macrophage infiltration, upregulation of pro-inflammatory cytokines (*IL-1β*, *IL-6*, *TNF-α*), and higher expression of atrophy-related genes (*FoxO3*, *MAFbx*, *MuRF1*) in skeletal muscle. Along with these changes, we also observed increased CK, LDH, and BUN levels in serum, which are usually regarded as biomarkers of EIMD. These findings are consistent with previous research [22,26,27]. Additionally, we compared the effects of STHI and LTMI training on exhaustive exercise-induced muscle damage. We discovered that STHI, in contrast to LTMI, increases muscle damage, suggesting that the weekend warrior approach might not protect against, and could even exacerbate, muscle damage after intense exercise. 

In both animal and human studies, exercise has been shown to cause alterations in gut microbiota composition and function, with the specific effects varying according to different exercise training modalities [28,29,30]. While the influence of exercise on gut microbiota is becoming clearer, the precise role of various exercise training patterns in shaping gut microbiota, particularly in the context of muscle damage following exhaustive exercise, remains not fully understood. In the present study, we showed that the richness and diversity of gut microbiota, as evidenced by the Chao and Shannon indices, were significantly different among the control group and two training groups. The increase in α-diversity, as indicated by the Chao and Shannon indices in the STHI group, was unexpected and contradicted much of the literature [31]. A previous study has shown that short-term, strenuous military training led to an increase in the Shannon index among young soldiers. This increase was attributed to the rise in abundance of less dominant taxa, including several potentially deleterious and infectious taxa such as *Staphylococcus*, *Peptoniphilus*, *Acidaminococcus*, and *Fusobacterium*. Concurrently, there was a decrease in more dominant taxa, such as *Bacteroides*. Consequently, an increase in the ratio of less-abundant, potentially harmful taxa to beneficial taxa may explain the unexpected observation that greater increases in the Shannon index during military training were correlated with larger increases in intestinal permeability [32]. Similarly, our study found that STHI training increased the abundance of less dominant taxa thought to promote inflammation, including *Staphylococcus* and *Bilophila*, and decreased the abundance of more dominant taxa, such as *norank_f: Muribaculaceae*. Moreover, the abundance of both *Staphylococcus* and *Bilophila* was positively correlated with inflammation levels following exhaustive exercise. Therefore, our findings may be interpreted in the context of the aforementioned study. While this provides a plausible explanation, we also acknowledge the possibility that other factors may contribute to the observed changes in α-diversity. The PCoA based on Bray–Curtis distances revealed that the gut microbiota composition significantly differed among the three groups. Furthermore, LEfSe analysis confirmed these differences, highlighting distinct taxonomic compositions at the phylum, family, and genus levels. Considering that SCFAs are a major class of bacterial metabolites, we further examined the levels of SCFAs in feces, including acetate, propionate, and butyrate. The results showed that LTMI training significantly increased fecal levels of SCFAs, including acetate, propionate, and butyrate, whereas STHI training did not result in statistically significant changes in these levels. Increasing research evidence suggests that gut microbiota and its metabolites, such as SCFAs, play an important role in the various physiological adaptations induced by exercise [33,34]. A previous study has found that changes in gut microbiota caused by aerobic exercise are associated with enhancements in exercise capacity and muscle mitochondrial energy metabolism [35]. Another study discovered that elite marathon runners exhibit a high abundance of *Veillonella* in their gut, and the propionate produced from the metabolism of lactate by this bacterium can enhance exercise endurance [36]. Therefore, it appears that gut microbiota and its SCFA metabolites are uniquely influenced by the two distinct training modalities, and these alterations may contribute to their contrasting effects on muscle damage induced by exhaustive exercise.

Mounting evidence demonstrates that gut microbiota and its SCFA metabolites are tightly correlated with inflammation under certain conditions [37,38]. In the present study, we conducted a correlation analysis to examine the relationship between muscle damage, indicated by the expression levels of inflammatory cytokines, and the presence of specific gut microbial communities at the genus level, as well as the levels of SCFAs. The results showed that the abundance of *Akkermansia*, *Prevotellaceae_NK3B31_group*, *Odoribacter*, *Alistipes*, and *Lactobacillus*, as well as the levels of SCFAs including acetate, propionate, and butyrate, were significantly negatively associated with the expression levels of inflammatory cytokines. In contrast, the abundance of *Staphylococcus* and *Bilophila* were notably positively associated with the expression levels of inflammatory cytokines. It is noteworthy that *Akkermansia*, *Prevotellaceae_NK3B31_group*, *Odoribacter*, *Alistipes*, and *Lactobacillus*, as major gut bacteria that produce SCFAs [39,40,41], have been demonstrated to have anti-inflammatory effects in many previous studies [42,43,44,45,46,47,48]. Furthermore, accumulating evidence suggests that gut microbiota-derived SCFAs are important mediators exerting various health benefits [49,50]. *Staphylococcus* and *Bilophila*, which are classified as opportunistic pathogens within the gut, have been shown to promote inflammation [51,52,53,54,55]. Collectively, our findings suggest that the protective effect of LTMI training on exhaustive exercise-induced muscle damage may be due to the increase in levels of SCFAs, which result from the increased abundance of certain SCFA-producing gut microbiota. Conversely, the worsening effect of STHI training on exhaustive exercise-induced muscle damage may be due to an increase in some opportunistic pathogens within the gut.

Fecal microbiota transplantation (FMT) serves as a valuable model in scientific research for understanding the physiological and pathological roles of gut microbiota [56,57]. In this study, we further clarified the role of gut microbiota alterations induced by two distinct training modalities in influencing muscle damage caused by exhaustive exercise via the method of FMT. We conducted FMT experiments using fecal samples from mice trained with different exercise patterns and assessed the impact on muscle damage caused by exhaustive swimming in recipient mice. Notably, mice receiving microbiota from mice trained in different modalities exhibited either reduced or exacerbated muscle damage following exhaustive swimming, compared to those receiving microbiota from sedentary controls. These results were in line with the findings from study 1, suggesting that FMT can replicate the effects of different exercise training patterns on muscle damage following exhaustive swimming. Yuan et al. [58] demonstrated that moderate-intensity aerobic exercise can lead to reductions in pathogenic bacteria such as intestinal *Allobaculum* and increases in probiotic bacteria such as *Akkermansia* in APP/PS1 transgenic Alzheimer’s disease (AD) mice. This modulation can reduce lipopolysaccharide (LPS) displacement and ultimately alleviate AD-related neuroinflammation. Exercise preconditioning with running wheels significantly enriched *Lactobacillus* and *Alistipes* in mice, a regulatory effect on gut microbiota that may contribute to the improvement of cognitive dysfunction after ischemic stroke by alleviating inflammation [59]. Lai et al. [60] reported that treadmill running (30 min/day, 5 days/week) led to a significant increase in *Odoribacter* abundance, which may contribute to decreased inflammation in diet-induced obese mice. It is noteworthy that the aforementioned microbiota are known producers of SCFAs, which can regulate several leukocyte functions, including the production of cytokines such as *TNF-α*, *IL-6*, and *IL-10*, eicosanoids, and chemokines, and affect the ability of leukocytes to migrate to the foci of inflammation and to destroy microbial pathogens [61]. In study 1, we found that LTMI training increased the abundance of *Akkermansia*, *Odoribacter*, *Alistipes*, and *Lactobacillus*, as well as the fecal levels of SCFAs, including acetate, propionate, and butyrate, and these changes were negatively correlated with the expression levels of certain inflammatory cytokines in mice following exhaustive exercise. Karl et al. [32] demonstrated that short-term, strenuous exercise, such as high-intensity cross-ski marching for four days, led to a decrease in the relative abundance of health-beneficial microbes and an increase in potentially inflammatory bacteria in soldiers. Specifically, the abundance of *Peptostreptococcus*, *Staphylococcus*, *Peptoniphilus*, *Acidaminococcus*, and *Fusobacterium* was found to increase. In study 1, we observed that STHI training increased the abundance of *Lachnospiraceae_NK4A136_group*, *Staphylococcus*, *Bacteroides*, *Roseburia*, and *Bilophila* in mice. Specifically, the changes in *Staphylococcus* and *Bilophila* were positively related to the expression levels of certain inflammatory cytokines following exhaustive exercise. You et al. [62] reported that traumatic brain injury (TBI)-induced gut microbiota dysbiosis may contribute to intestinal inflammation by altering bile acid profiles, with specific bacterial taxa such as *Staphylococcus* and *Lachnospiraceae* being implicated. Furthermore, in mice fed a high-fat diet, increased inflammation was associated with the increased abundance of bacterial genera, including *Oscillospira*, *Desulfovibrio*, *Coprobacillus*, *Enterococcus*, *Intestinimonas*, *Blautia*, and *Bilophila*. In contrast, intervention with pistachio and polyphenol-rich oolong tea decreased the abundance of these bacteria, thereby improving inflammation [63,64]. Together with the findings from FMT experiment, it can be inferred that alterations in gut microbiota contribute to the opposing effects of LTMI and STHI training on skeletal muscle damage induced by exhaustive exercise. 

Although our results suggest that LTMI and STHI training regimens may have opposite effects on muscle damage following exhaustive exercise, potentially mediated by changes in gut microbiota, we acknowledge that this conclusion has limitations. The association analysis in our study was conducted with a relatively low animal sample size, which may limit the reproducibility or generalizability of our findings. Additionally, the FMT experiments, while providing valuable insights into exploring the mediator effect of the gut microbiota, would provide stronger evidence when combined with fecal 16S rRNA sequencing in the recipient mice after FMT. Furthermore, we cannot infer that the “weekend warrior” exercise pattern would exacerbate muscle damage after exhaustive exercise. Although our study’s STHI training regimen increased exercise intensity and condensed training time in a short period, similar to the “weekend warrior” pattern, it was not fully matched with the “weekend warrior” exercise pattern.

In summary, the two distinct exercise training modalities, LTMI training and STHI training, both with an equivalent total exercise volume, resulted in different alterations to gut microbiota and its SCFA metabolites and, respectively, mitigated and exacerbated muscle damage following exhaustive exercise in mice. The changes in gut microbiota caused by the two training patterns may have contributed, at least partly, to their opposing effects on exhaustive exercise-induced muscle damage. Our findings highlight the importance of a proper training mode prior to participating in exhaustive exercise. The STHI training regimen shares some similarities with the “weekend warrior” pattern commonly observed in practice, such as promoting exercise intensity and condensing training sessions into a short period. However, this approach may not be an optimal strategy for preparing athletes for prolonged strenuous exercises, such as marathon running. 

## 4. Materials and Methods

### 4.1. Animals

Seven-week-old male C57BL/6J mice (7 weeks, 19–21 g) were purchased from the Laboratory Animal Centre of the Army Medical University (Chongqing, China). All mice were housed in a controlled environment (22 °C–25 °C room temperature, 50–55% relative humidity, 12 h light/dark cycle) and fed on a chow diet and distilled water ad libitum for 1 week of acclimation to the environment before the experiment began.

### 4.2. Ethics Statement

The animal experiment protocol was approved by the Ethics Committee of the Army Medical University. Animal care was performed in accordance with the China Laboratory Animal Management Regulations and the Guide for the Care and Use of Laboratory Animals (Institute for Laboratory Animal Research, Washington, DC, USA).

### 4.3. Experiment Design

**Figure 7:**
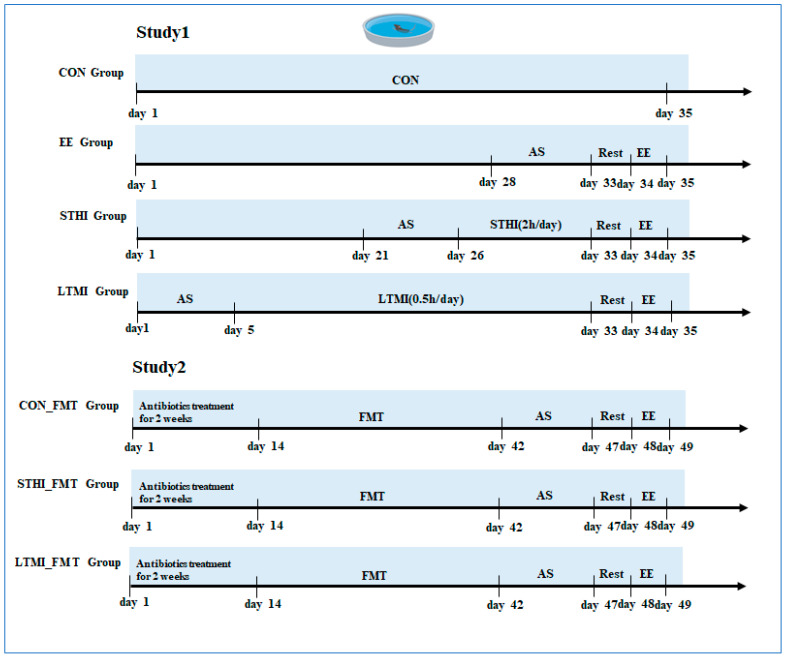
A schematic of the experimental schedule. AS, adaptive swimming; CON, control; EE, exhaustive exercise; STHI, short-term, high-intensity training; LTMI, long-term, moderate-intensity training; CON_FMT, transplantation of fecal microbiota of sedentary mice in study 1; STHI_FMT, transplantation of fecal microbiota of short-term, high-intensity training mice in study 1; LTMI_FMT, transplantation of fecal microbiota of long-term, moderate-intensity training mice in study 1.

Study 1: Thirty-two mice were randomly divided into four groups with eight mice each: sedentary group (control, CON), exhaustive exercise group (EE), exhaustive exercise after short-term, high-intensity training group (STHI-EE), and exhaustive exercise after long-term, moderate-intensity training group (LTMI-EE). Mice in the control group remained in resting conditions in the cage. Mice in the other groups performed adaptive swimming for 10 min per day for 5 days before the corresponding intervention. Mice in the STHI-EE group were subjected to continuous swimming for 2 h once a day in the morning for 1 week, whereas mice in the LTMI-EE group were subjected to swimming for 30 min once a day in the morning for 4 weeks, totaling 14 h of swimming training in both groups. Swimming was conducted in a round tank (100 cm in diameter, 50 cm deep) filled with water, and the water temperature was kept at about 35 °C controlled by a thermostat. To address instances of the animals ceasing active swimming, we employed a soft brush to gently stimulate the water surface near the mice, encouraging them to resume swimming. This non-invasive technique is minimal and is in line with the ethical treatment of animals in research [65]. Before undergoing the exhaustive swimming, the mice were rested for one day. Exhaustion was determined when the mice remained submerged for 5 s and showed a blunted righting reflex according to the Thomas and Marshall criteria [65]. A schematic of the experimental schedule is presented in Figure 7.

Stool samples from all groups were collected on day 34. On day 35, the mice of the control group were sacrificed in the morning, and the mice of the other groups were sacrificed immediately after exhaustive swimming. Blood samples were collected rapidly from the orbital venous plexus and centrifuged at 1500× *g* for 15 min to collect the serum. The gastrocnemius tissue was collected, a part of tissue was fixed in 4% paraformaldehyde for histopathological examination, and the rest of tissue was immediately stored in liquid nitrogen and then transferred to a −80 °C refrigerator.

Study 2 (fecal microbiota transplantation): FMT was performed through oral gavage with a fecal microbiota suspension, a method that has been extensively utilized in animal studies [66,67]. After one week of acclimation to the environment, seven-week-old C57BL/6J male recipient mice were randomly divided into 3 groups with 6 mice each: transplantation of fecal microbiota of sedentary mice in study 1 (CON_FMT), transplantation of fecal microbiota of STHI training mice in study 1 (STHI_FMT), and transplantation of fecal microbiota of LTMI training mice in study 1 (LTMI_FMT). The endogenous gut microbiota of recipient mice was depleted by treatment with an antibiotic combination of ampicillin (1 g/L, A830931; Macklin Biochemical Technology, Shanghai, China), vancomycin (0.5 g/L, V105495, Aladdin Biochemical Technology, Shanghai, China), metronidazole (1 g/L, M109874, Aladdin Biochemical Technology, Shanghai, China), and neomycin (1 g/L, N109017, Aladdin Biochemical Technology, Shanghai, China) in their drinking water for 2 weeks. Then, the recipient mice received a total oral dose of 200 μL of corresponding FMT inoculations once daily for 4 weeks. After transplantation, all mice were familiarized with swimming for 10 min per day for 5 days. Subsequently, all the mice in the three groups were rested for one day and then subjected to exhaustive swimming. After the experiment, the mice were sacrificed by administering anesthesia, and blood and gastrocnemius tissues were collected for further analyses. A schematic of the experimental schedule is also presented in Figure 7.

### 4.4. Histopathological Analyses

The muscle tissue from the gastrocnemius was first preserved in a solution of 4% paraformaldehyde (Beyotime Biotechnology, Shanghai, China). Following this, the tissue underwent dehydration using a series of ethanol washes before being embedded in paraffin. Subsequently, the embedded tissue was cut into sections with a thickness of 5 μm. Prior to staining, the sections were treated to remove paraffin and rehydrate the tissue through a graded ethanol to xylene series. Hematoxylin and Eosin (H&E) staining was then performed to visualize the tissue structure. The stained sections were examined using a Leica DM750 light microscope (Leica Microsystems, Wetzlar, Germany). For documentation, images were captured with a 20× objective lens and recorded using the Leica MC170 HD digital imaging system.

### 4.5. Immunofluorescence Staining

The slides were incubated with primary antibodies against F4/80 (1:100, AB204467; Abcam, Cambridge, UK) and iNOS (1:20, AB3523, Abcam, Cambridge, UK) at 4 °C overnight. The slides were then incubated with a secondary antibody for 1 h at 4 °C. The images of the immunofluorescence-stained sections were obtained with a 20× objective using the Leica MC170 HD digital camera.

### 4.6. Serum Biochemical Parameters

LDH, CK, and BUN in the serum were measured by an automatic biochemical analyzer (2110, Hitachi, Japan).

### 4.7. Real-Time Quantitative PCR

Total RNA was extracted from the gastrocnemius homogenate using RNAiso Plus reagent (Takara Bio, Kusatsu, Japan) according to the manufacturer’s instructions. The RNA EEcentration and purity were assessed by a NanoDropTM 2000 spectrophotometer (Thermo Fisher Scientific Inc., Waltham, MA, USA). Total RNA was reverse transcribed to cDNA using a PrimeScript RT Master Mix kit (Takara Bio, Shiga, Japan) in accordance with the manufacturer’s instructions. qRT-PCR was carried out with the qTower 2.2 real-time PCR system (Anakytik Jena, Thuringia, Germany) using SYBR Premix Ex Taq II (Takara Bio, Japan). The primers for the targeted genes were synthesized by Sangon Biotech (Shanghai, China). The primer sequences used for gene expression analysis are listed in Table 1. The PCR protocol consisted of denaturation at 95 °C for 30 s, followed by 40 cycles of 95 °C for 5 s and 60 °C for 30 s. A melting curve protocol was run at the end of the amplification. Relative fold changes in gene expression were analyzed by the 2^−ΔΔCt^ method using GAPDH as an internal control gene. 

### 4.8. Western Blotting

The muscle samples were homogenized in RIPA Lysis Buffer and centrifuged at 10,000× *g* (10 min, 4 °C) to obtain total protein. Then, the BCA Protein Assay Kit (Aidlab Biotechnologies Co., Ltd., Beijing, China) was used to quantify the supernatant protein concentrations in the extracts. Next, equal amounts of protein (50 μg per lane) were separated through 10% SDS-polyacrylamide gels and subsequently electrotransferred onto polyvinylidene fluoride (PVDF) membranes (Millipore, Marlborough, MA, USA). Then, the membranes were incubated with blocking buffer (1× TBS, 0.1% Tween 20, and 5% skim milk) at room temperature for 3 h, and then incubated with the primary antibodies (1:1000 dilutions with PBST) at 4 °C overnight: anti-IL-1β (1:1000, 26048-1-AP; Proteintech, Rosemont, IL, USA), anti-TNF-α (1:1000, 17590-1-AP; Proteintech, USA), anti-MuRF1 (1:1000, 55456-1-AP; Proteintech, USA), anti-MAFbx (1:1000, 67172-1-Ig; Proteintech, USA), anti-GAPDH (1:10,000, 60004-1-Ig; Proteintech, USA). The subsequent day, the membranes were exposed to secondary antibodies that were linked with horseradish peroxidase (HRP) for a duration of one hour at ambient temperature (1:1000 dilutions with PBST). Blots were detected using an enhanced chemiluminescence detection system (ChemiDoc MP, BIO-RAD, Hercules, CA, USA). 

### 4.9. Measurement of the Oxidative Stress Biomarkers

A total of 1 mL of extraction solvent was added to 100 mg of gastrocnemius samples and homogenized. Then the homogenate was centrifuged at 14,000× *g* for 10 min, and the supernatant was collected for measurement. The supernatant was assayed spectrophotometrically for malondialdehyde (MDA) and protein carbonyl (PC) content with commercial kits (BC0025, BC1275, respectively, Solarbio Science & Technology, Beijing, China). All procedures were performed in accordance with the manufacturer’s instructions.

### 4.10. Fecal Microbiome Analysis

The genomic microbial DNA from stool samples was extracted using a DNA Stool kit (PF Mag-Bind Stool DNA Kit, Norcross, GA, USA). The V3-V4 hypervariable region of 16S ribosomal RNA was chosen as the PCR amplified region by an ABI GeneAmp^®^ 9700 PCR thermocycler (Thermo Fisher Scientific, CA, USA). The bacterial forward primer was 338F (5′-ACTCCTACGGGAGGCAGCAG-3′) and the reverse primer was 806R (5′-GGACTACHVGGGTWTCTAAT-3′). The genes were sequenced on the Illumina MiSeq platform (Illumina, San Diego, CA, USA). The original sequences were qualitatively controlled using fastp software [68] and spliced using FLASH software [69]. Operational taxonomic units (OTUs) with 97% similarity cutoff were clustered using UPARSE (v.11) [70,71], and chimeric sequences were identified and removed. Based on the ASVs information, rarefaction curves and alpha diversity indices, including observed ASVs, Chao1 richness, Shannon index and Good’s coverage, were calculated with Mothur v1.30.2 [72]. Principal coordinate analysis (PCoA) based on Bray–Curtis dissimilarity was completed using the Vegan v2.4.3 package. The raw reads were deposited into the NCBl Sequence Read Archive (SRA) database (Accession Number: SRP509306).

We used R and STAMP (v.2.0.0) to identify the distinguishing taxa of the gut microbiota profiles of different groups [73]. We identified microbiota-based biomarkers using the Linear Discriminant Analysis Effect Size (LEfSe) method, which indicates that a linear discriminant analysis (LDA) score of >2.0 (false discovery rate (FDR) < 0.5) contributes to the uniqueness of the corresponding population.

### 4.11. Measurement of Fecal SCFAs

Frozen fecal samples were thawed on ice. A total of 50 mg of each sample and 1 mL methanol were added into a 2 mL polypropylene centrifuge tube with vortex mixing for 5 min to extract the SCFAs. Then, the mixture was centrifuged at 14,000× *g* for 10 min, and the supernatant was collected for measurement. Quantification of SCFAs was performed by high-performance liquid chromatography (HPLC) in supernatants reconstituted in PBS. Briefly, 1 mL of supernatant solution was acidified with 1/10 volume of H_2_SO_4_ (0.01 M) and passed through a condenser to isolate volatile compounds within a sample. Following filtration through a 0.45 μm membrane, an equal volume of samples was loaded onto the HPLC (Thermo Fisher U3000 liquid chromatograph); chromatographic column: C18 AQ (4.6 × 250 mm). Sulfuric acid (0.01 M) was used as the mobile phase, and the levels of SCFAs were determined by external standard calibration method. 

### 4.12. Statistical Analysis

The data are expressed as mean  ±  standard deviation. Statistical analysis was per-formed using GraphPad Prism 8.0.2(263) (Insightful Science, San Diego, CA, USA). Student’s *t*-test was utilized to compare two groups and differences between the multigroup were determined by ANOVA with Bonferroni’s post hoc test. Correlation analysis was conducted using the Pearson method with R software (v.3.6.2). A *p*-value of less than 0.05 was set as the threshold for statistical significance. Asterisks indicate the level of significance: (ns, no significance; * *p* < 0.05; ** *p* < 0.01; *** *p* < 0.001; **** *p* < 0.0001).

## Figures and Tables

**Table 1:** Sequences of Primers Used in the RT-qPCR Analysis.

Gene Name	Species	Forward Primers Sequence (5′–3′)	Reverse Primers Sequence (5′–3′)
*IL-1β*	mouse	CCCAAGCAATACCCAAAGAA	TTGTGAGGTGCTGATGTACCA
*IL6*	mouse	GAACAACGATGATGCACTTGC	CTTCATGTACTCCAGGTAGCTATGGT
*TNF-α*	mouse	CTTCTGTCTACTGAACTTCGGG	CACTTGGTGGTTTGCTACGAC
*FoxO3a*	mouse	TCACTGTATTCAGCTAGTGCAA	ATGATGGACTCCATGTCACATT
*MAFbx*	mouse	GACTGGACTTCTCGACTGCC	TCAGGGATGTGAGCTGTGAC
*MuRF1*	mouse	ACCTGCTGGTGGAAAACATC	CTTCGTGTTCCTTGCACATC
*GPADH*	mouse	CATCACTGCCACCCAGAAGACTG	ATGCCAGTGAGCTTCCCGTTCAG

Abbreviations: *IL-1β*, Interleukin-1 beta; *IL-6*, Interleukin-6; *TNF-α*, Tumor Necrosis Factor alpha; *FoxO3*, Fork-head box O3; *MAFbx*, Muscle Atrophy F-box; *MuRF1*, muscle-specific RING Finger Protein 1; *GAPDH*, glyceraldehyde-3-phosphate dehydrogenase.

## Data Availability

The original contributions presented in the study are included in the article. Further inquiries can be directed to the corresponding author.
